# Taohong Siwu Decoction alleviates high salt-induced calcium overload and ferroptosis in vascular endothelial cells in hypertension by regulating ATF4

**DOI:** 10.3389/fnut.2025.1647017

**Published:** 2025-09-11

**Authors:** Lai Kwan Lam, Peng-Li Xu, Peng-Cheng Xie, Qiu-er Liang, Ting Xie, Lee Yam Poon, Ya Xiao, Li-Guo Chen

**Affiliations:** ^1^School of Traditional Chinese Medicine, Jinan University, Guangzhou, China; ^2^Department of Obstetrics and Gynaecology, Faculty of Medicine, The Chinese University of Hong Kong, Hong Kong SAR, China; ^3^The Third Affiliated Hospital, Guangzhou University of Traditional Chinese Medicine, Guangzhou, China; ^4^Affiliated Dongguan People's Hospital, Southern Medical University, Dongguan, China; ^5^Center of Integrated Chinese and Western Medicine, The First Affiliated Hospital of Xiamen University, School of Medicine, Xiamen University, Xiamen, China

**Keywords:** Taohong Siwu Decoction, hypertension, vascular endothelial injury, ATF4, calcium overload, ferroptosis

## Abstract

**Background:**

Taohong Siwu Decoction (THSWD), a traditional Chinese medicine formula, is increasingly applied in clinical practice for hypertension management. Our previous research demonstrated that THSWD alleviates high-salt-induced hypertension in mice. This study aims to further elucidate the underlying mechanisms of THSWD in treating hypertension.

**Methods:**

The chemical composition of THSWD was identified using UPLC-Q/TOF-MS in earlier research. In this study, we performed both *in vivo* and *in vitro* experiments. *ATF4^+/−^* mice (KO) and C57BL/6 mice (WT) were fed a high-salt diet with or without THSWD treatment. Human aortic endothelial cells (HAECs) were cultured in high-NaCl conditions, with or without ATF4 inhibition. Blood pressure, vascular injury, calcium overload, and ferroptosis were measured to evaluate the protective effects of THSWD.

**Results:**

*In vivo*, a high-salt diet caused hypertension, vascular wall thickening, vascular injury, calcium overload, and ferroptosis, all of which were significantly alleviated by THSWD and the calcium-channel blocker nifedipine (NI). THSWD also reduced the high-salt-induced overexpression of ATF4. Similar effects were observed *in vitro*, where THSWD, the ferroptosis inhibitor ferrostatin-1 (Fer-1), the intracellular calcium chelator BAPTA-AM, and NI improved calcium overload and ferroptosis caused by high-NaCl. This was accompanied by reduced expression of CaMK4, CACNA1C, IP3R, RyR2, GPX4, ACSL4, and LPCAT3. Furthermore, compared to *ATF4^+/−^* mice on a high-salt diet, those treated with THSWD showed greater reductions in blood pressure, improved vascular endothelial function, and better suppression of calcium overload and ferroptosis. Inhibition of ATF4 or co-treatment with siATF4 and THSWD *in vitro* also restored abnormal biomarker levels (iron, calcium, 12-HETE, 15-HETE, GSH, GSH/GSSG, MDA, and LPO) and normalized calcium overload- and ferroptosis-related markers.

**Conclusion:**

THSWD effectively lowers blood pressure and protects against vascular damage in high-salt-induced hypertension. Its protective effects are achieved by inhibiting calcium overload and ferroptosis through the regulation of ATF4.

## Graphical abstract

The proposed mechanism of THSWD in the treatment of high salt-induced hypertension and vascular endothelial injury. 
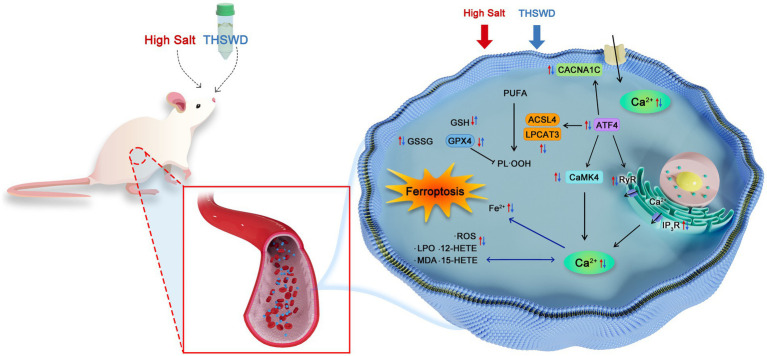

## 1 Introduction

Hypertension is defined by elevated pressure within blood vessels, with systolic blood pressure reaching or exceeding 140 mmHg and/or diastolic blood pressure reaching or exceeding 90 mmHg. It is classified into primary and secondary hypertension. Primary hypertension occurs when the underlying cause of high blood pressure is not identifiable (1). In contrast, secondary hypertension results from medical conditions such as renal and adrenal disorders (2). The global aging population and persistent unhealthy lifestyle choices, including physical inactivity and high sodium consumption, have contributed to a rising incidence of hypertension (3, 4). The prevalence of hypertension remains high worldwide. According to the World Health Organization’s 2019 Global Hypertension Report, 1.3 billion people globally have been diagnosed with hypertension, and one-third of adults have elevated blood pressure (5).

Hypertension has become a major global public health issue, directly linked to heart failure, kidney disease, left ventricular hypertrophy, carotid atherosclerosis, peripheral vascular disease, and subclinical cardiovascular disease (6). The goal of hypertension treatment is to achieve optimal blood pressure levels and reduce the incidence of associated cardiovascular events (7). The primary medical strategy involves the ongoing use of antihypertensive medications, including angiotensin II receptor blockers, angiotensin-converting enzyme inhibitors, diuretics, and calcium channel blockers (8). These drugs have proven effective in lowering blood pressure, preventing complications, and reducing mortality among hypertensive individuals. Non-pharmacological recommendations emphasize healthy eating, lifestyle modifications, regular physical activity, and limited alcohol consumption (9). Despite these efforts, the management and prevention of hypertension continue to face significant challenges globally.

Salt-sensitive hypertension (SSHTN) represents a critical phenotype characterized by blood pressure fluctuations in response to dietary sodium intake (10). Salt intake is directly linked to high blood pressure and represents a substantial risk factor for hypertension and cardiovascular complications. Excessive salt consumption leads to a notable increase in blood pressure and salt sensitivity, particularly affecting individuals sensitive to salt (11–13). This condition affects approximately 25% of normotensive individuals and over 50% of hypertensive patients (14), posing a substantial public health challenge. The global mean sodium intake is approximately 4,310 mg/day, far exceeding the recommended 2,000 mg/day by World Health Organization (15). Evidence indicates a correlation between high salt intake and ferroptosis in hypertension. Ferroptosis, a type of non-apoptotic and iron-dependent cell death, occurs when cells experience iron overload and the lipid peroxidation repair system, which relies on glutathione (GSH), is compromised. This process leads to the accumulation of reactive oxygen species (ROS) and triggers cell death (16). Driven by oxidative stress and lipid peroxidation, ferroptosis accelerates vascular endothelial cell (VEC) aging and injury, while protective autophagy mitigates these effects, playing a key role in endothelial dysfunction (17). Another study demonstrated that ferroptosis contributes to vascular endothelial dysfunction through iron-catalyzed lipid peroxidation, glutathione depletion, and disruption of cellular antioxidant systems, resulting in membrane damage and organelle dysfunction (18). The ferroptosis inhibitor ferrostatin-1 (Fer-1) has shown potential in reducing elevated blood pressure in high-salt diet-induced hypertension models (19). However, the role of THSWD in regulating ferroptosis in hypertension, particularly through the ATF4 pathway, remains largely unexplored.

Activating transcription factor 4 (ATF4), characterized by a basic leucine zipper structure, regulates genes involved in the unfolded protein response (UPR) (20) and the expression of multiple target genes such as C/EBP-homologous protein (CHOP) and Death receptor 5 (DR5) through the PERK-eIF2α-ATF4 pathway involved in endoplasmic reticulum stress (ERS) (21). It is related to endothelial dysfunction in hypertension. However, the precise mechanism underlying the ferroptosis of vascular endothelial cells due to a high-salt diet remains uncertain. Excessive ROS generated by iron can significantly impact calcium (Ca^2+^) entry into cells and the maintenance of intracellular Ca^2+^ storage, disrupting Ca2 + homeostasis (22). This forms a vicious cycle, as excessive Ca^2+^ levels subsequently result in higher iron levels (23). Furthermore, lipid peroxidation products like 4-hydroxynonenal (4-HNE) can stimulate membrane Ca^2+^ channels, and elevated levels of 4-HNE can increase Ca^2+^ levels by reducing the function of Na^+^-Ca^2+^ pumps and modifying the permeability of Ca^2+^ channels (24). Proteins involved in the Ca^2+^ signaling pathway, such as ryanodine receptor 2 (RyR2) and inositol 1,4,5-trisphosphate receptors (IP3R), are sensitive to redox regulation (25). Increased Ca^2+^ uptake by mitochondria can lead to the generation of ROS and raised levels of Fe^2+^, both of which can result in ferroptosis (26).

Taohong Siwu Decoction (THSWD) has a long history of use in treating hypertension, with established benefits in reducing blood stasis and improving circulation (27). The formula consists of six herbs: *Prunus persica* (L.) Batsch (family Rosaceae), Angelica sinensis (Oliv.) Diels (family Apiaceae), *Carthamus tinctorius* L. (family Asteraceae), Ligusticum Chuangxiong Hort. (family Apiaceae), Rehmannia glutinosa (Gaertn.) DC. (family Orobanchaceae), and *Paeonia lactiflora* Pall. (family Paeoniaceae). A meta-analysis on the efficacy of THSWD combined with antihypertensive drugs suggested it has better antihypertensive effects than drugs alone (28). Previous studies showed that THSWD effectively enhances endothelial cell activity and significantly protects endothelial cells (29). THSWD-serum promoted the growth of human umbilical vein endothelial cells (HUVECs), activated nitric oxide synthase (eNOS), and enhanced nitric oxide (NO) secretion (30). Our studies found that THSWD reduced blood pressure in spontaneously hypertensive rats (31) and ameliorated hypertensive nephropathy by inhibiting ferroptosis (19). Network pharmacology results indicated that the calcium signaling pathway is a main pathway for THSWD in treating and preventing hypertension (32). However, although preliminary studies have been conducted on THSWD’s effects on hypertension, the specific mechanisms by which THSWD alleviates hypertension remain unexplored.

## 2 Methods

### 2.1 Preparation of THSWD for *in vivo* experiment

The dosage and preparation methods of THSWD utilized in this study were consistent with those outlined in our earlier research (19, 33), and we conducted UPLC-Q/TOF-MS analysis of THSWD, with detailed information available in our previous publication (33). In order to streamline the process of quality control, all ingredients in THSWD were sourced from the first affiliated hospital of Jinan University. These ingredients included The formula of THSWD consists of six herbs: *Prunus persica* (L.) Batsch, Angelica sinensis (Oliv.) Diels, *Carthamus tinctorius* L., Ligusticum Chuangxiong Hort., Rehmannia glutinosa (Gaertn.) DC., and *Paeonia lactiflora* Pall. The Chinese herbs composition ratio is as previous study: 3: 3: 2: 2: 4: 3 (*Prunus persica* (L.): Angelica sinensis (Oliv.) Diels: *Carthamus tinctorius* L.: Ligusticum Chuangxiong Hort.: Rehmannia glutinosa (Gaertn.) DC.: Paeonia lactiflora Pall.).

### 2.2 Animals and treatment

The wild-type mice were purchased from Guangdong GemPharmatech Co., Ltd., with the strain being C57BL/6 J; *ATF4^+/−^* mice were purchased from Jiangsu GemPharmatech Co., Ltd. The SPF level 6–8 week old *ATF4^+/−^* mice and C57BL/6 J mice used in the study were bred at the Experimental Animal Center of Jinan University. The animal experimental protocol was approved by the Institutional Animal Care and Use Committee of Jinan University (approval number: IACUC-20220923-07), and all animal experiments were supervised by the Medical Ethics Committee of Jinan University and conducted in accordance with the guide for the care and use of laboratory animals published by NIH (#85–23, revised in 1985).

A total of 30 C57BL/6 mice were randomly divided into 5 group: normal diet group (ND), high salt diet group (HSD), high salt diet+low dose of Taohong Siwu decoction group (HSD + THSWD-L), high salt diet+high dose of Taohong Siwu decoction group (HSD + THSWD-H), and high salt diet+nifedipine group (HSD-NI), with 6 mice in each group. A total of 24 *ATF4*^+/−^ mice were randomly divided into 4 groups: *ATF4^+/−^* with normal diet group (ND-KO), *ATF4^+/−^*with high salt diet group (HSD-KO), *ATF4^+/−^* with high salt diet and low dose of Taohong Siwu decoction group (HSD + THSWD-L-KO), and *ATF4^+/−^* with high salt diet and high dose of Taohong Siwu decoction group (HSD + THSWD-H-KO), with 6 mice in each group.

The high-salt diet group was fed with 8% high-salt diet for 8 weeks to establish a hypertension mice model. The drug groups continued to receive a high-salt diet and were administered Taohong Siwu decoction orally, while the nifedipine group was given nifedipine (34), throughout the study period of 4 weeks of the interventions.

### 2.3 Human cell lines

The concentration of high NaCl used in this study aligns with that described in our previous research, with detailed information provided in our earlier publication (35). The human aortic endothelial cells (HAECs) (#6100) were purchased from Sciencell in the United States. They were adherent cells with spindle-shaped morphology. The cells were cultured in high-glucose DMEM (Gibco, USA) medium containing 10% fetal bovine serum (FBS) (Gibco, USA) and 1% penicillin–streptomycin (Gibco, USA), and maintained at 37.0°C with a CO_2_ concentration of 5% in a cell culture incubator. When the confluence of HAECs reached over 80%, they were passaged using trypsin (2.5% EDTA) (Gibco, USA). NaCl 155 mM (Sigma, USA), 2.5% THSWD-containing serum, 3.75% THSWD-containing serum, ferrostatin-1 (Fer-1) 4 μM (MedChemExpress, USA), BAPTA-AM 2.5 μM (MedChemExpress, USA) and nifedipine (NI) 4 μM (MedChemExpress, USA) were used to incubated HAECs for 24 h. An osmotic control was used to further verify the high NaCl concentrations in the experimental setup (Supplementary Figure 1).

### 2.4 Measurement of blood pressure

The blood pressure of mice in each group was measured using the BP-2,000 Blood Pressure Analysis System (Visitech System, USA). During the measurement process, the temperature of the detection platform was maintained at 37 °C, with a quiet surrounding environment to ensuring stable emotions of mice. The mice were fixed on the detection platform and their numbers input into the Blood Pressure Analysis program. The base of each mouse’s tail was be passed through a sensor and then fixed with medical tape to ensure that its body and tail are in a straight line. The first measurement was performed before the high-salt diet, the rest of measurements for mice were conducted every 2 weeks by the same operator.

### 2.5 Detection of endothelial secretion of factors

The endothelial secretion of factors was evaluated by measuring the levels of nitric oxide (NO), endothelin 1 (ET-1) and vascular endothelial growth factor (VEGF) levels in the serum of mice. The levels of NO, ET-1, and VEGF levels in serum of mice were determined using corresponding enzyme-linked immunosorbent assay (ELISA) kits (MM-0658 M1, MM-0561 M1, MM-0128 M1, Jiangsu Meimian, China) according to the manufacturer’s instructions.

### 2.6 Hematoxylin–eosin staining

The aorta tissues of mice were washed with physiological saline to remove blood stains and excess tissue on the arterial surface, then dried with filter paper. The tissues were immediately fixed in 4% paraformaldehyde at room temperature. The mouse aortic tissues underwent the following steps: dehydration, embedding, sectioning, dewaxing of paraffin sections to water, hematoxylin–eosin (HE) staining, and dehydration sealing. Using Nikon DS-U3 microscope imaging software (Nikon, Japan), the purposeful areas of the tissue were imaged at 100x and 400x magnification using the lower right corner scale as a reference point. Imagej 1.54 software (National Institutes of Health, USA) was used to measure the thickness of the vessel wall.

### 2.7 Transmission electron microscopy

The aorta tissues of mice were collected and immediately fixed in electron microscope fixative for 2 h at 4 °C. After fixed with 1% osmium tetroxide and dehydrated, the ultrathin sections were stained with 2% uranyl acetate and lead citrate. The ultrastructure of aorta tissues was visualized using a transmission electron microscope (Hitachi, Japan).

### 2.8 Detection of iron

Iron level in mouse aortas or HAECs were detected using iron colorimetric assay kit (ADS-W-D007, Aidisheng, China), following the manufacturer’s instructions. The mouse aortas or HAECs were homogenized in iron assay buffer. After centrifugation, the 10% of the supernatant was carefully collected. Absorbance was read at a wavelength of 562 nm using a microplate reader (Biotech, Germany).

### 2.9 Detection of calcium

Calcium (Ca^2+^) level in mouse aortas or HAECs were detected using calcium microplate assay kit (S05312-1, Meimian, China), following the manufacturer’s instructions. The mouse aortas or HAECs were homogenized in calcium assay buffer. After centrifugation, the 10% of the supernatant was carefully collected. Absorbance was read at a wavelength of 610 nm using a microplate reader.

### 2.10 Detection of ferroptosis biomarkers

12-Hydroxyeicosatetraenoic acid (12-HETE), 15-Hydroxyeicosatetraenoic acid (15-HETE), malondialdehyde (MDA) and lipid peroxidation (LPO) in mouse aortas were determined by using corresponding ELISA kits (MM-0695 M1, MM-45461 M1, MM-0897 M1, MM-0716 M1, Meimian, China). And glutathione (GSH) and glutathione disulfide (GSSG) in mouse aortas and HAECs were determined by using GSH and GSSG assay kit (A061-1, Jiancheng Bioengineering, China). 12-HETE, 15-HETE, MDA, LPO and glutathione peroxidase 4 (GPX4) in HAECs were determined by corresponding ELISA kits (MM-60353H1, MM-51630H1, MM-2037H1, MM-1378H1, Meimian, China). All detections were conducted strictly in accordance with the instructions of the reagent kit. Absorbance was read at a wavelength of 450 nm using a microplate reader.

### 2.11 Detection of cell vitality

Cell Counting Kit-8 (CCK-8) (CK04, Dojindo, Japan) was used to measure cell viability. HAECs were treated with a medium containing different concentration of NaCl, THSWD-containing serum, Fer-1, BAPTA-AM and NI were used to incubated HAECs at 37 °C for 24 h. Atfer transfering the liquid from each well, 10 μL of CCK-8 solution was added and incubated in a CO2 incubator for 1 h. Microplate reader was used to detect the absorbance at a wavelength of 450 nm.

### 2.12 Cell transfection

Small interfering RNA (siRNA) transfection was performed with siATF4 (Human) and negative control siRNA (RiboBio, China). HAECs were seeded equally in 6-well plates at a confluency of 50% and transfected with the RiboFect™ CP transfection kit (C10511-05, RiboBio, China), followed by an incubation at 37 °C for 24 h. The concentrations of siRNAs were 30 nM.

### 2.13 Detection of reactive oxygen species

Reactive oxygen species (ROS) fluorometric assay kit (E-BC-K138-F, Elabscience, China) was used to measure ROS levels in HAECs. Using the reagent to treat HAECs according to the instructions, DCFH-DA reagent was added and incubated at 37 °C for 30 min to 1 h. After removing the DCFH-DA reagent, HAECs were digested with trypsin to prepare a cell suspension. HAECs were collected and resuspended in corresponding reagent, then were detected using flow cytometer (Agilent technologies, USA) and analyze the data using FlowJo (FlowJo, USA).

### 2.14 Live-cell calcium imaging

Live-cell calcium imaging was performed to detect to intracellular calcium levels in HAECs by confocal laser microscopy (Zeiss, Germany) using Fluo-4 Calcium Assay Kit (S1061S, Beyotime, China). Using the reagent to treat HAECs according to the instructions, Fluo-4 reagent was added and incubated at 37 °C for 30 min. After the incubation, observation under a laser confocal microscope. Imagej 1.54 software was used for image analysis.

### 2.15 Extraction of RNA and real-time quantitative PCR

Total RNA from mouse aortas or HAECs was extracted using TRIzol (DP424, Tiangen, China), followed by cDNA synthesis with the RevertAid First Strand cDNA Synthesis Kit (K1622, Thermo, USA). Real-time quantitative PCR (RT-qPCR) was performed to quantify the mRNA expression using 2x SYBR Green qPCR Master Mix (B21203, Selleck, USA) by CFX96™ real-time system (Bio-Rad, USA). The relative expression level change of the target gene was calculated using 2-ΔΔCt based on the Ct values of the samples. The PCR primers for all the genes analyzed were listed in Supplementary Tables S1, S2.

### 2.16 Western blotting

The RIPA buffer (high) (R0010, Solarbio, USA) was used to lyse the mouse aortas or HAECs in order to extract protein for further experiments. Protein quantification detections of the lysis supernatant were performed using the BCA Protein Assay Kit (P0011, Beyotime, China). The protein samples underwent separation through SDS polyacrylamide gel electrophoresis (SDS-PAGE) using SDS-PAGE Gel Kit (P1200, Solarbio, USA) and were transferred into a polyvinylidene fluoride (PVDF) membrane (IPVH00010, Millipore, Germany). Following closure and washing of the PVDF membranes, they were incubated with a primary antibody for 20 h, followed by a 2-h incubation with a secondary antibody. The protein on the PVDF membranes were processed and developed using BeyoECL Plus (ECL like Western reagent) (P0018S, Beyotime, China) to enhance chemiluminescence. The antibodies used for western blotting were ATF4 (11815S, CST), calcium/Calmodulin Dependent Protein Kinase IV (CaMK4) (4032S, CST), calcium voltage-gated channel subunit alpha1 (CACNA1C) (ab84814, Abcam), IP3R (8568S, CST), RyR2 (ab302716, Abcam), GPX4 (59735S, CST), acyl-CoA synthetase long chain family member 4 (ACSL4) (ab155282, Abcam), lysophosphatidylcholine Acyltransferase 3 (LPCAT3) (ab232958, Abcam), glyceraldehyde-3-phosphate dehydrogenase (GAPDH) (2118S, CST).

### 2.17 Statistical analysis

The data were analyzed using GraphPad Prism 8 software (GraphPad Software, USA), with quantitative data presented as mean±standard deviation. One-way analysis of variance (ANOVA) was employed for comparing means between multiple groups with post-hoc Fisher’s LSD test or Tukey’s *post hoc* test. The Student’s t-test was employed to assess the presence of significant differences between two groups. *p* < 0.05 indicated a statistically significant difference.

## 3 Results

### 3.1 THSWD improves hypertension and vascular endothelial dysfunction

**Figure 1:**
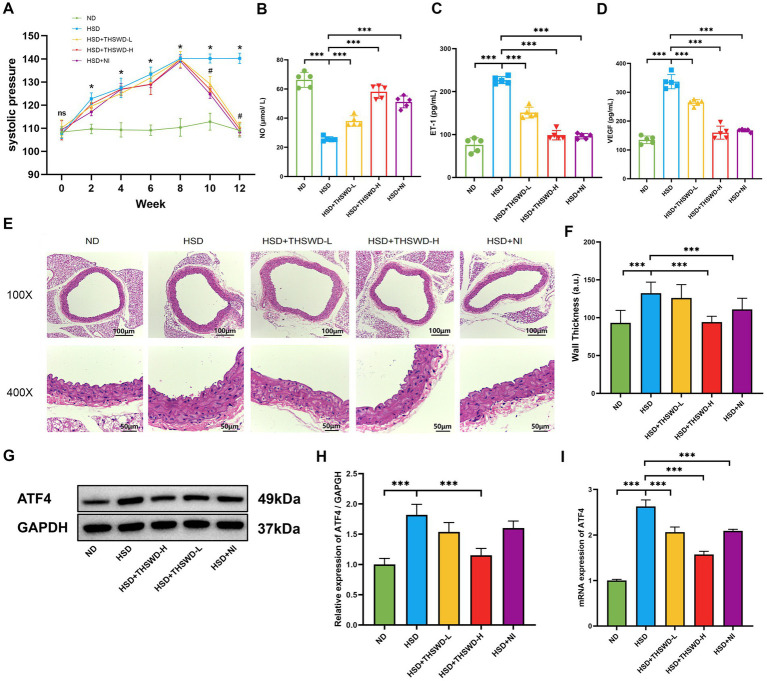
THSWD improved blood pressure, vascular injury biomarkers and the expression of ATF4 in high-salt diet mice. **(A)** Systolic pressure. Data was presented as mean ± SEM (*n* = 6, per group), *p < 0.05 vs. ND group, #*p* < 0.05 vs. HSD group. **(B)** NO levels **(C)** ET-1 levels **(D)** VEGF levels. Data was presented as mean ± SEM (*n* = 5, per group). *p < 0.05, **p < 0.01, ***p < 0.001. **(E)** HE staining of aortic tissues in mice. **(F)** Arterial wall thickness based on HE staining. Data was expressed as mean ± SEM (*n* = 10, per group). *p < 0.05, **p < 0.01, ***p < 0.001. **(G,H)** Western blotting results of ATF4. Data was presented as mean ± SEM (*n* = 3, per group), *p < 0.05 vs. HSD group. **(I)** RT-qPCR results of ATF4. Data was presented as mean ± SEM (*n* = 3, per group). *p < 0.05, **p < 0.01, ***p < 0.001. ND, normal diet; HSD, high salt diet; HSD + THSWD-L, high salt diet with low-dose Taohong Siwu decoction; HSD + THSWD-H, high salt diet with high-dose Taohong Siwu decoction; HSD + NI, high salt diet with nifedipine.

The results indicated that the increase of blood pressure of mice in the high-salt diet group exhibited a positive correlation with the duration of high-salt exposure, reaching its peak at the 8th week compared to mice in the normal diet group (*p* < 0.05). Mice fed with a high-salt diet showed a decreasing trend in blood pressure after receiving high-dose THSWD, low-dose THSWD, and nifedipine treatment starting from the 9th week, approaching the blood pressure level of the mice in normal group by the 12th week, compared to the HSD group mice, there was a significant decrease in blood pressure (*p* < 0.05) (Figure 1A). NO level were lower and ET-1, VEGF levels were higher in mice fed a high-salt diet (*p* < 0.001). Reversal of these alterations was observed with high-dose THSWD, low-dose THSWD, and nifedipine treatment, leading to a increase in NO levels and decrease in ET-1 and VEGF levels (*p* < 0.001), and high-dose THSWD had a more potent effect (Figures 1B–D). Pathological staining of aortic tissues unveiled morphological changes induced by hypertension resulting from the high-salt diet. HE staining showed significant morphological changes in blood vessels, with disordered arrangement of strands, uneven and discontinuous inner walls, and increased vessel thickness (*p* < 0.001). With the treatment of high-dose THSWD and nifedipine, the vascular morphology improved, and the thickness of blood vessels decreased significantly (*p* < 0.001) (Figures 1E,F).

The results of Western blot and qRT-PCR showed that a high-salt diet led to an increase in protein and mRNA expression of ATF4 (*p* < 0.001). After treatment with high-dose THSWD, the protein and mRNA expression levels of ATF4 decreased (*p* < 0.001). And after treatment with low-dose THSWD and nifedipine, the mRNA expression levels of ATF4 decreased to varying degrees (*p* < 0.001). THSWD downregulated the protein and mRNA expression of ATF4 in a dose-dependent manner (Figures 1G–I).

### 3.2 THSWD suppresses calcium overload in high-salt-induced hypertensive mice

**Figure 2:**
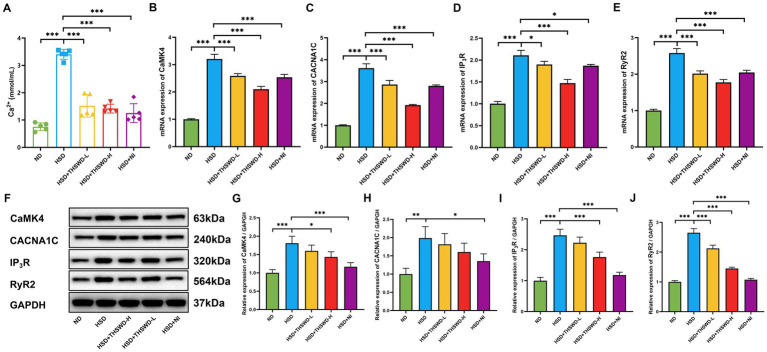
THSWD attenuated calcium overload in high-salt diet mice. **(A)** Calcium levels. Data was presented as mean ± SEM (n = 5, per group). *p < 0.05, **p < 0.01, ***p < 0.001. **(B–E)** RT-qPCR results of CaMK4, CACNA1C, IP3R and RyR2. Data was presented as mean ± SEM (*n* = 3, per group). *p < 0.05, **p < 0.01, ***p < 0.001. **(F–J)** Western blotting results of CaMK4, CACNA1C, IP3R and RyR2. Data was presented as mean ± SEM (*n* = 3, per group). *p < 0.05, **p < 0.01, ***p < 0.001. ND, normal diet; HSD, high salt diet; HSD + THSWD-L, high salt diet with low-dose Taohong Siwu decoction; HSD + THSWD-H, high salt diet with high-dose Taohong Siwu decoction; HSD + NI, high salt diet with nifedipine.

High-salt diet induced an increase in Ca^2+^ level in aorta of hypertensive mice (*p* < 0.001). High-dose THSWD, low-dose THSWD, and nifedipine significantly reversed increase in Ca^2+^ level (*p* < 0.001) (Figure 2A). High-salt diet induced high protein and mRNA expression of CaMK4, CACNA1C, IP3R, and RyR2 (*p* < 0.01; *p* < 0.001), THSWD significantly downregulated both protein and mRNA expression of CaMK4, IP3R, and RyR2, as well as the mRNA expression of CACNA1C (*p* < 0.05; *p* < 0.001). Similarly, nifedipine downregulated the protein and mRNA expression of CaMK4, CACNA1C, IP3R, and RyR2 (*p* < 0.05; *p* < 0.001) (Figures 2B–J). Low-dose THSWD also downregulated the mRNA expression of CaMK4, CACNA1C, IP3R, and RyR2, and the protein expression of RyR2 (*p* < 0.05; *p* < 0.001). The inhibitory capacity of High-dose THSWD on CaMK4, CACNA1C, IP3R, and RyR2 was more prominent.

### 3.3 THSWD suppresses ferroptosis in high-salt-induced hypertensive mice

**Figure 3:**
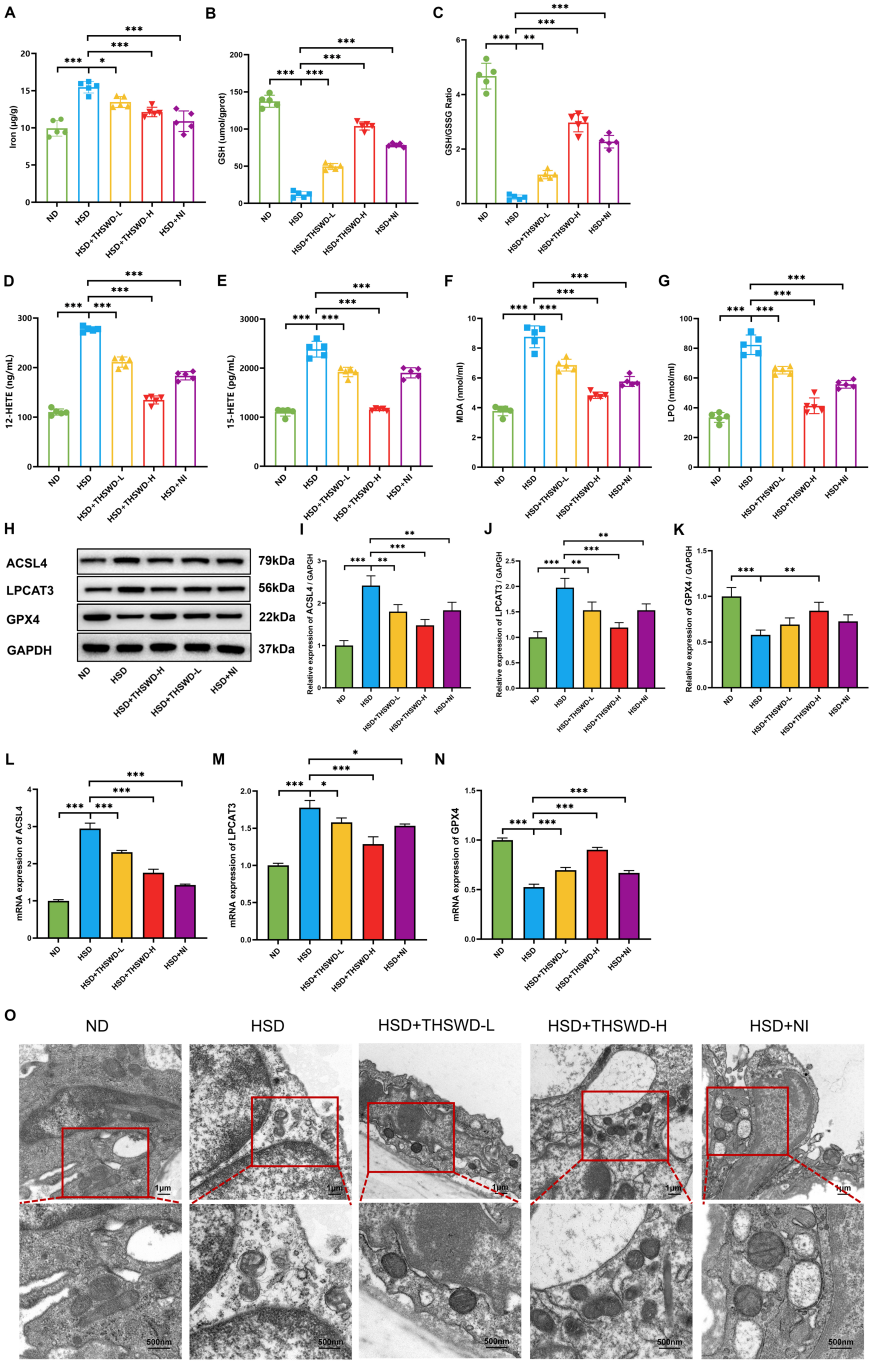
THSWD attenuated ferroptosis in high-salt diet mice. **(A)** Iron levels. **(B)** GSH levels. **(C)** GSH/GSSG levels. **(D)** 12-HETE levels. **(E)** 15-HETE levels. **(F)** MDA levels. **(G)** LPO levels. Data was presented as mean ± SEM (*n* = 5, per group). *p < 0.05, **p < 0.01, ***p < 0.001. **(H–K)** Western blotting results of ACSL4, LPCAT3 and GPX4. Data was presented as mean ± SEM (*n* = 3, per group). *p < 0.05, **p < 0.01, ***p < 0.001. **(L–N)** RTqPCR results of ACSL4, LPCAT3 and GPX4. Data was presented as mean ± SEM (*n* = 3, per group). *p < 0.05, **p < 0.01, ***p < 0.001. **(O)** Transmission electron microscopy analysis of aorta tissue. Scale bars, 1 μm and 500 nm. ND, normal diet; HSD, high salt diet; HSD + THSWD-L, high salt diet with low-dose Taohong Siwu decoction; HSD + THSWD-H, high salt diet with high-dose Taohong Siwu decoction; HSD + NI, high salt diet with nifedipine.

High-salt diet-induced calcium overload in the aorta in mice mitigated by THSWD and nifedipine. In order to further explore the mechanism of THSWD, we conducted measurements on ferroptosis-related biomarkers. In hypertensive mice induced by a high-salt diet, there were elevated levels of iron, 12-HETE, 15-HETE, MDA, and LPO, and reduced the levels of GSH and GSH/GSSG (*p* < 0.001). Treatment with high-dose THSWD, low-dose THSWD, and nifedipine reversed these levels (*p* < 0.05; *p* < 0.01; *p* < 0.001), with high-dose THSWD having the most significant impact on ferroptosis-related biomarkers (Figures 3A–G).

In mice with high-salt-induced hypertension, there was a significant increase in the protein and mRNA expression of ACSL4 and LPCAT3, along with a decrease in GPX4 expression (*p* < 0.001). Treatment with both doses of THSWD and nifedipine resulted in downregulation of ACSL4 and LPCAT3 protein and mRNA expression (*p* < 0.05; *p* < 0.01; *p* < 0.001). High-dose THSWD also upregulated GPX4 protein and mRNA expression (*p* < 0.01; *p* < 0.001) (Figures 3H,N). The inhibitory effects of high-dose THSWD on ACSL4, LPCAT3, and GPX4 were more pronounced.

Furthermore, electron microscopy showed that high-dose THSWD, low-dose THSWD, and nifedipine effectively reversed mitochondrial swelling, deformation, and cristae damage in mouse aortic endothelial cells caused by a high-salt diet (Figure 3O).

### 3.4 THSWD-containing serum suppresses calcium overload and ferroptosis in HAECs exposed to high NaCl

**Figure 4:**
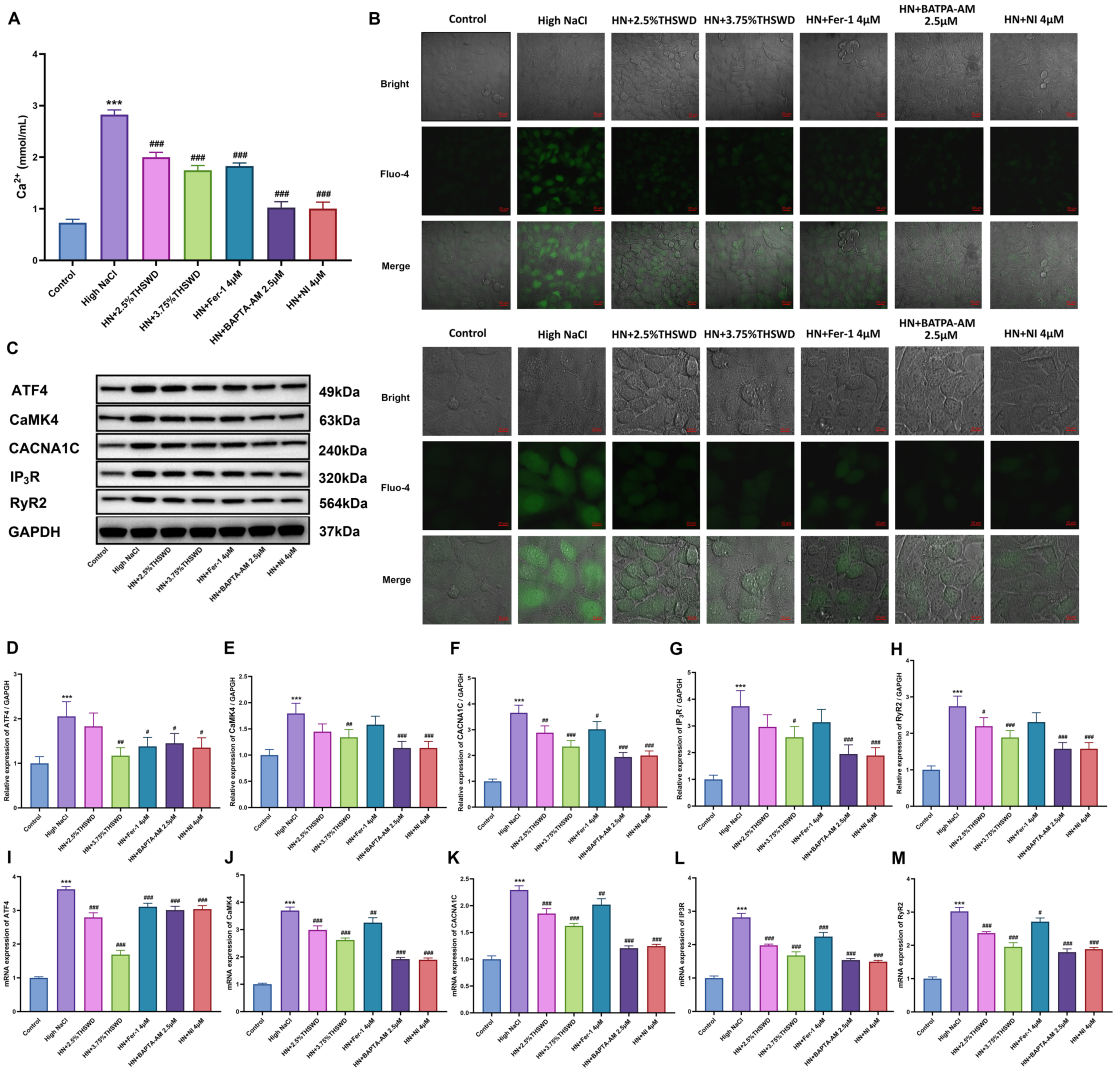
THSWD-containing serum reduced calcium overload in HAECs. **(A)** Calcium levels. Data was presented as mean ± SEM (*n* = 6, per group). #*p* < 0.05, ##*p* < 0.01, ###*p* < 0.001 vs. Control group; *p < 0.05, **p < 0.01, ***p < 0.001 vs. High NaCl group. **(B)** Confocal fluo-4-Calcium images. Scale bars, 20 μm and 10 μm. **(C-H)** Western blotting results of CaMK4, CACNA1C, IP3R and RyR2. Data was presented as mean ± SEM (*n* = 3, per group). #*p* < 0.05, ##*p* < 0.01, ###*p* < 0.001 vs. Control group; *p < 0.05, **p < 0.01, ***p < 0.001 vs. High NaCl group. **(I–M)** RT-qPCR results of CaMK4, CACNA1C, IP3R and RyR2. Data was presented as mean ± SEM (*n* = 3, per group). #*p* < 0.05, ##*p* < 0.01, ###*p* < 0.001 vs. Control group; *p < 0.05, **p < 0.01, ***p < 0.001 vs. High NaCl group. Control, normal group; High NaCl, High NaCl group; HN + 2.5%THSWD, High NaCl+low concentration of THSWD-containing serum group; HN + 3.75%THSWD, High NaCl+high concentration of THSWD-containing serum group; HN + Fer-1 4 μM, High NaCl+ferrostatin-1 group; HN + BAPTA-AM 2.5 μM, High NaCl+BAPTA-AM group; HN + NI 4 μM, High NaCl+nifedipine group.

Exposure of HAECs to high NaCl significantly elevated intracellular calcium levels. This elevation was significantly attenuated when cells were incubated with 2.5% or 3.75% THSWD-containing serum, Fer-1, BAPTA-AM, or NI, resulting in decreased Ca^2+^ levels (*p* < 0.001) (Figure 4A). Fluo-4, a fluorescent dye, indicates cellular Ca^2+^ concentrations, producing bright green fluorescence when bound to Ca^2+^. HAECs exposed to high NaCl exhibited strong green fluorescence, which was attenuated when cells were incubated with 2.5 and 3.75% THSWD-containing serum, Fer-1, BAPTA-AM, and NI (Figure 4B). High NaCl exposure increased the protein and mRNA expression of ATF4, CaMK4, CACNA1C, IP3R, and RyR2 (*p* < 0.001). When cells were incubated with 3.75% THSWD-containing serum, BAPTA-AM, or NI, the protein and mRNA expression of ATF4, CaMK4, CACNA1C, IP3R, and RyR2 was significantly downregulated (*p* < 0.05; *p* < 0.01; *p* < 0.001). Additionally, incubation with 2.5% THSWD-containing serum reduced the protein and mRNA expression of CACNA1C, RyR2N (*p* < 0.05; *p* < 0.01; *p* < 0.001). Fer-1 also downregulated the mRNA expression of these genes (*p* < 0.05; *p* < 0.01; *p* < 0.001) (Figures 4C–M). The inhibitory effects of 3.75% THSWD-containing serum, BAPTA-AM, and NI on CaMK4, CACNA1C, IP3R, and RyR2 were particularly notable.

**Figure 5:**
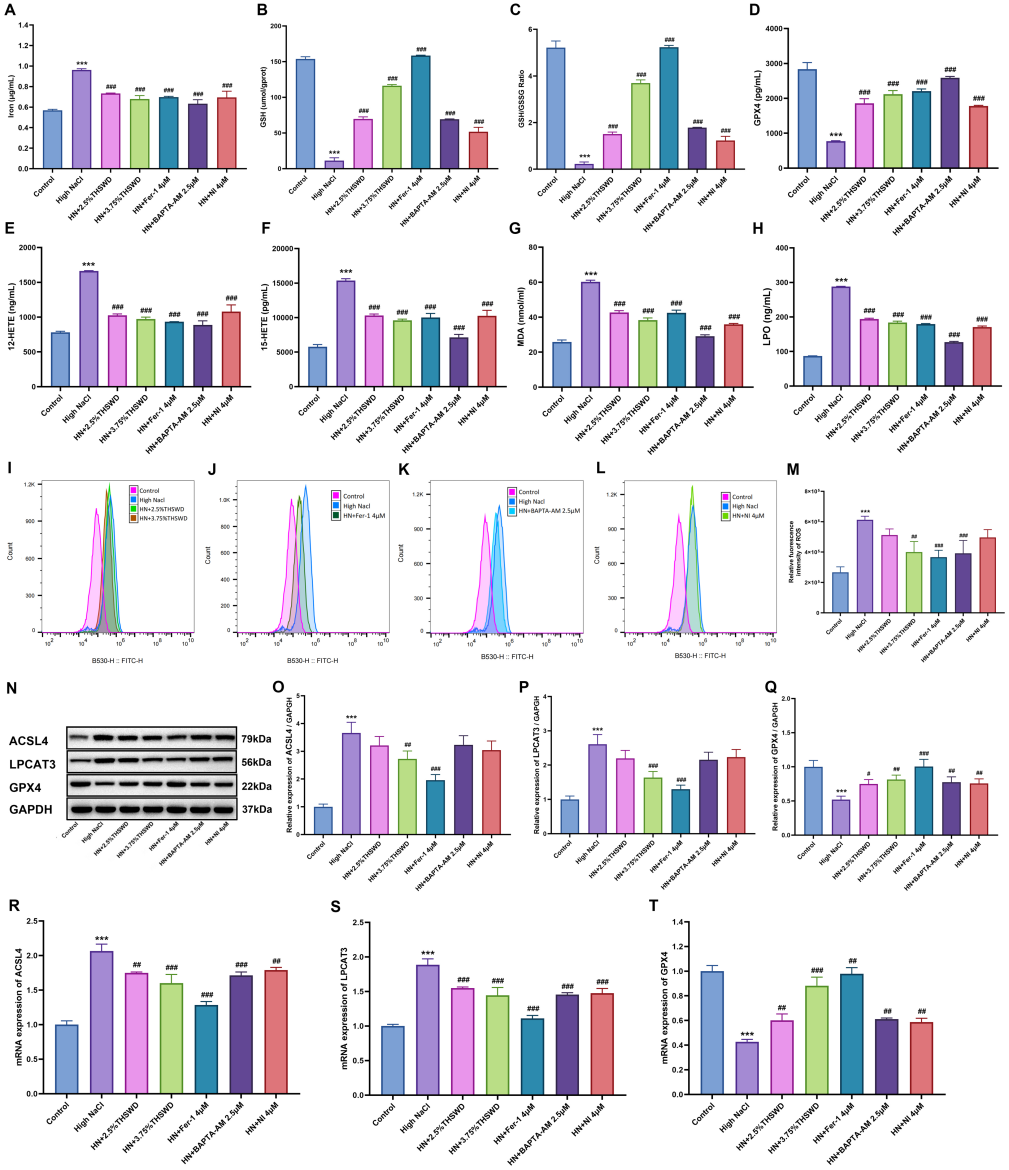
THSWD-containing serum reduced ferroptosis in HAECs. **(A)** Iron levels. **(B)** GSH levels. **(C)** GSH/GSSG levels. **(D)** GPX4 levels. **(E)** 12-HETE levels. **(F)** 15-HETE levels. **(G)** MDA levels. **(H)** LPO levels. Data was presented as mean±SEM (*n* = 3, per group). #*p* < 0.05, ##*p* < 0.01, ###*p* < 0.001 vs. Control group; *p < 0.05, **p < 0.01, ***p < 0.001 vs. High NaCl group. **(I–M)** ROS levels. Data was presented as mean ± SEM (*n* = 3, per group). #*p* < 0.05, ##*p* < 0.01, ###*p* < 0.001 vs. Control group; *p < 0.05, **p < 0.01, ***p < 0.001 vs. High NaCl group. **(N–Q)** Western blotting results of ACSL4, LPCAT3 and GPX4. Data was presented as mean ± SEM (*n* = 3, per group). #*p* < 0.05, ##*p* < 0.01, ###*p* < 0.001 vs. Control group; *p < 0.05, **p < 0.01, ***p < 0.001 vs. High NaCl group. **(R–T)** RT-qPCR results of ACSL4, LPCAT3 and GPX4. Data was presented as mean ± SEM (*n* = 3, per group). *p < 0.05, **p < 0.01, ***p < 0.001 vs. Control group; #*p* < 0.05, ##*p* < 0.01, ###*p* < 0.001 vs. High NaCl group. Control, normal group; High NaCl, High NaCl group; HN + 2.5%THSWD, High NaCl+low concentration of THSWD-containing serum group; HN + 3.75%THSWD, High NaCl+high concentration of THSWD-containing serum group; HN + Fer-1 4 μM, High NaCl+ferrostatin-1 group; HN + BAPTA-AM 2.5 μM, High NaCl+BAPTA-AM group; HN + NI 4 μM, High NaCl+nifedipine group.

Significant increases were observed in the levels of iron, 12-HETE, 15-HETE, MDA, and LPO, while GPX4, GSH and GSH/GSSG levels decreased in HAECs exposed to high NaCl (*p* < 0.001). When cells were incubated with 2.5 and 3.75% THSWD-containing serum, Fer-1, BAPTA-AM, and NI, the aberrant levels of iron, GPX4, GSH, GSSG, 12-HETE, 15-HETE, MDA, and LPO caused by high NaCl were effectively reversed (*p* < 0.001) (Figures 5A–H). Additionally, ROS detection results showed that high NaCl significantly increased ROS levels in HAECs (*p* < 0.001). This increase was significantly reduced when cells were incubated with 3.75% THSWD-containing serum, Fer-1, and BAPTA-AM (*p* < 0.01; *p* < 0.001) (Figures 5I–M). High NaCl exposure also led to increased protein and mRNA expression of ACSL4 and LPCAT3, along with decreased expression of GPX4 (*p* < 0.001). Incubation with 3.75% THSWD-containing serum and Fer-1 significantly reduced the protein and mRNA levels of ACSL4 and LPCAT3, while increasing GPX4 expression (*p* < 0.01; *p* < 0.001). Similarly, incubation with 2.5% THSWD-containing serum, BAPTA-AM, and NI decreased the mRNA expression of ACSL4 and LPCAT3 and increased GPX4 protein and mRNA expression (*p* < 0.01; *p* < 0.001) (Figures 5N–T). The inhibitory effects of 3.75% THSWD-containing serum and Fer-1 on ACSL4, LPCAT3, and GPX4 were particularly notable.

### 3.5 ATF4 deficiency improves vascular endothelial dysfunction and enhances THSWD’S effects in high-salt-induced *ATF4^+/−^* mice

**Figure 6:**
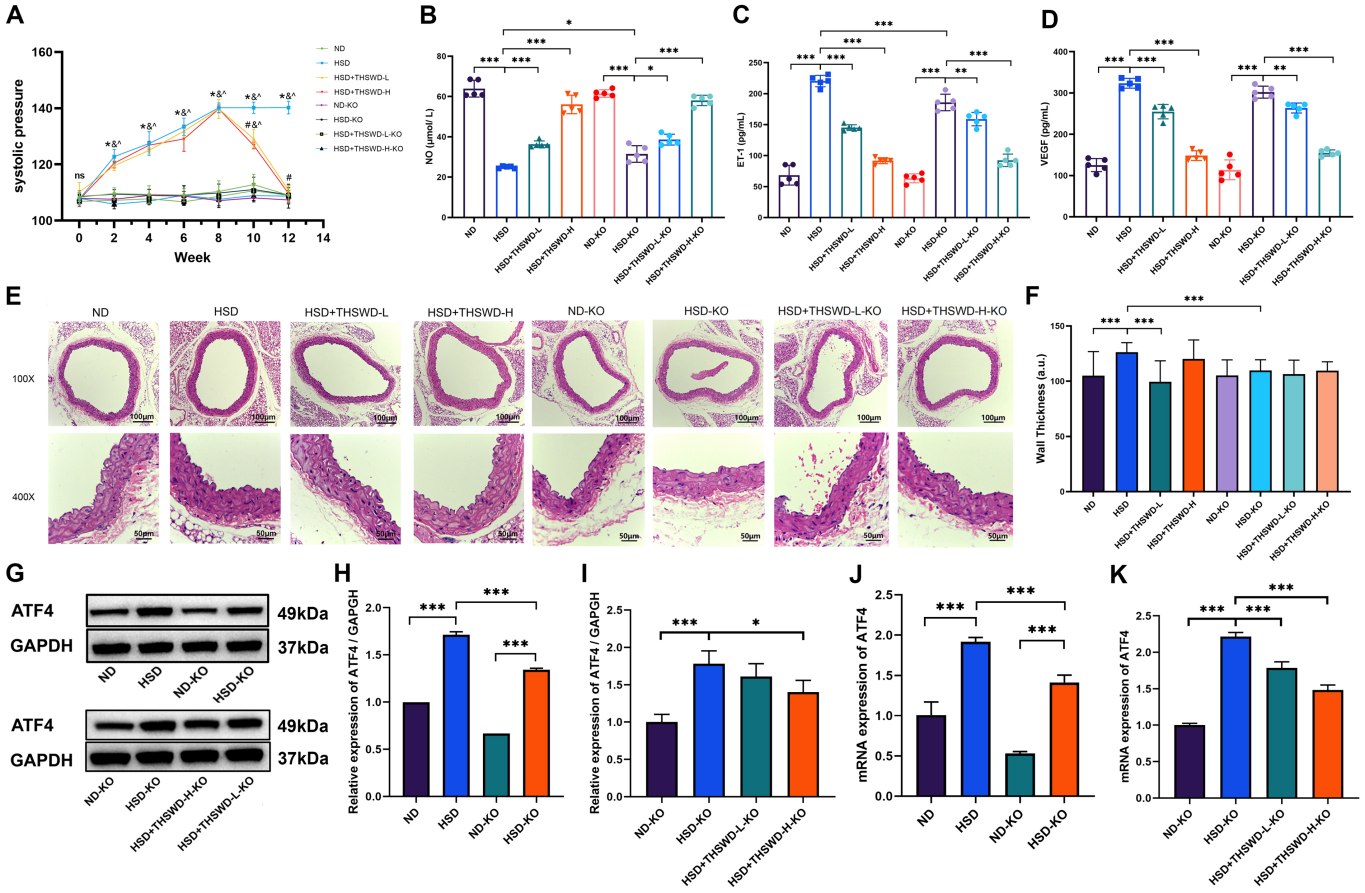
ATF4 knockdown and ATF4 knockdown plus THSWD treatment improved blood pressure, vascular injury biomarkers and the expression of ATF4 in high-salt diet mice. **(A)** Systolic pressure. Data was presented as mean ± SEM (*n* = 6, per group). *p < 0.05, **p < 0.01, ***p < 0.001. **(B)** NO levels **(C)** ET-1 levels **(D)** VEGF levels. Data was presented as mean ± SEM (*n* = 5, per group). *p < 0.05, **p < 0.01, ***p < 0.001. **(E)** HE staining of aortic tissues in mice. **(F)** Arterial wall thickness based on HE staining. Data was expressed as mean ± SEM (*n* = 10, per group), *p < 0.05 vs. HSD group. **(G-I)** Western blotting results of ATF4. Data was presented as mean ± SEM (*n* = 3, per group). *p < 0.05, **p < 0.01, ***p < 0.001. **(J-K)** RTqPCR results of ATF4. Data was presented as mean±SEM (n = 3, per group), *p < 0.05 vs. HSD group. ND, wild-type+normal diet group; HSD, wild-type+high salt diet group; HSD + THSWD-L, wild-type+high salt diet+low-dose Taohong Siwu decoction group; HSD + THSWD-H, wild-type+high salt diet+high-dose Taohong Siwu decoction group; ND-KO, *ATF4^+/−^* + normal diet group; HSD-KO, *ATF4^+/−^* + high salt diet group; HSD + THSWD-L-KO, *ATF4^+/−^* + high salt diet+low-dose Taohong Siwu decoction group; HSD + THSWD-H-KO: ATF*4^+/−^* + high salt diet+high-dose Taohong Siwu decoction group.

To investigate the downstream effects, we established an *ATF4^+/−^* mouse model and conducted experiments involving a high-salt diet following treatment with both high-dose and low-dose THSWD. ATF4 knockdown significantly reduced blood pressure (*p* < 0.05), with no notable differences observed among the ATF4 knockdown groups, even after interventions with varying doses of THSWD (Figure 6A). The knockdown also effectively elevated NO levels and decreased ET-1 levels (*p* < 0.05; *p* < 0.001). Varying doses of THSWD further enhanced the levels of NO, and reduced the levels of ET-1 and VEGF (*p* < 0.05; *p* < 0.01; *p* < 0.001) (Figures 6B–D).

ATF4 knockdown effectively preserved vascular morphology and significantly reversed the increase in vascular wall thickness induced by a high salt diet (*p* < 0.001) (Figures 6E,F). Additionally, ATF4 protein and mRNA expression levels were reduced in ATF4 knockdown mice (*p* < 0.001), validating the model’s effectiveness. High-dose THSWD further downregulated ATF4 protein and mRNA expression levels (*p* < 0.05; *p* < 0.001), whereas low-dose THSWD also decreased ATF4 mRNA expression (*p* < 0.001) (Figures 6G–K).

### 3.6 ATF4 deficiency inhibits calcium overload and ferroptosis enhancing THSWD’S regulation in *ATF4^+/−^* mice

**Figure 7:**
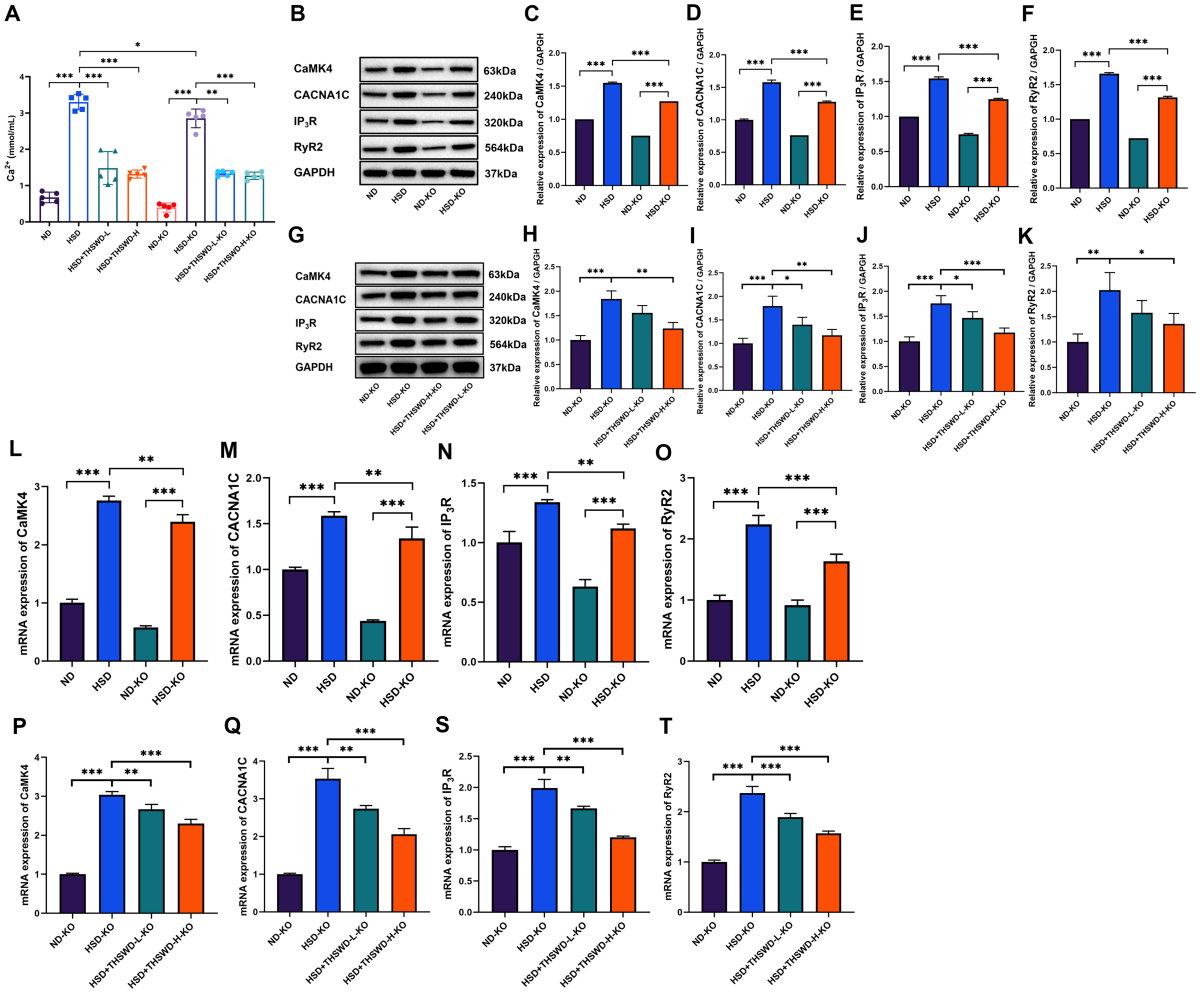
ATF4 knockdown and ATF4 knockdown plus THSWD treatment attenuated calcium overload in high-salt diet mice. **(A)** Calcium levels. Data was presented as mean±SEM (n = 5, per group). *p < 0.05, **p < 0.01, ***p < 0.001. **(B–F)** Western blotting results of CaMK4, CACNA1C, IP3R and RyR2 in ND, HSD, ND-KO and HSD-KO groups. Data was presented as mean ± SEM (*n* = 3, per group). *p < 0.05, **p < 0.01, ***p < 0.001. **(G–K)** Western blotting results of CaMK4, CACNA1C, IP3R and RyR2 in ND-KO, HSD-KO, HSD + THSWD-L-KO and HSD + THSWD-H-KO groups. Data was presented as mean ± SEM (*n* = 3, per group). *p < 0.05, **p < 0.01, ***p < 0.001. **(L–O)** RTqPCR results of CaMK4, CACNA1C, IP3R and RyR2 in ND, HSD, ND-KO and HSD-KO groups. Data was presented as mean ± SEM (*n* = 3, per group). *p < 0.05, **p < 0.01, ***p < 0.001. **(P–T)** RT-qPCR results of CaMK4, CACNA1C, IP3R and RyR2 in ND-KO, HSD-KO, HSD + THSWD-L-KO and HSD + THSWD-H-KO groups. Data was presented as mean±SEM (*n* = 3, per group). *p < 0.05, **p < 0.01, ***p < 0.001. ND, wild-type+normal diet group; HSD, wild-type+high salt diet group; HSD + THSWD-L, wild-type+high salt diet+low-dose Taohong Siwu decoction group; HSD + THSWD-H, wild-type+high salt diet+high-dose Taohong Siwu decoction group; ND-KO, *ATF4^+/−^* + normal diet group; HSD-KO, *ATF4^+/−^* + high salt diet group; HSD + THSWD-L-KO, *ATF4^+/−^* + high salt diet+low-dose Taohong Siwu decoction group; HSD + THSWD-H-KO: ATF*4^+/−^* + high salt diet+high-dose Taohong Siwu decoction group.

ATF4 knockdown significantly decreased aortic calcium levels (*p* < 0.05). Following treatment with both high-dose and low-dose THSWD, a further reduction in aortic calcium levels was observed (*p* < 0.01; *p* < 0.001) (Figure 7A). The knockdown effectively inhibited the elevation of CaMK4, CACNA1C, IP3R, and RyR2 protein and mRNA expression levels (*p* < 0.01; *p* < 0.001) (Figures 7B–F,L). High-dose THSWD downregulated the expression of CaMK4, CACNA1C, IP3R, and RyR2 proteins and mRNA (*p* < 0.05; *p* < 0.01; *p* < 0.001). Additionally, low-dose THSWD reduced CACNA1C and IP3R protein and mRNA expression levels, as well as the mRNA expression of CaMK4 and RyR2 (*p* < 0.05; *p* < 0.01; *p* < 0.001) (Figures 7G–K,P).

**Figure 8:**
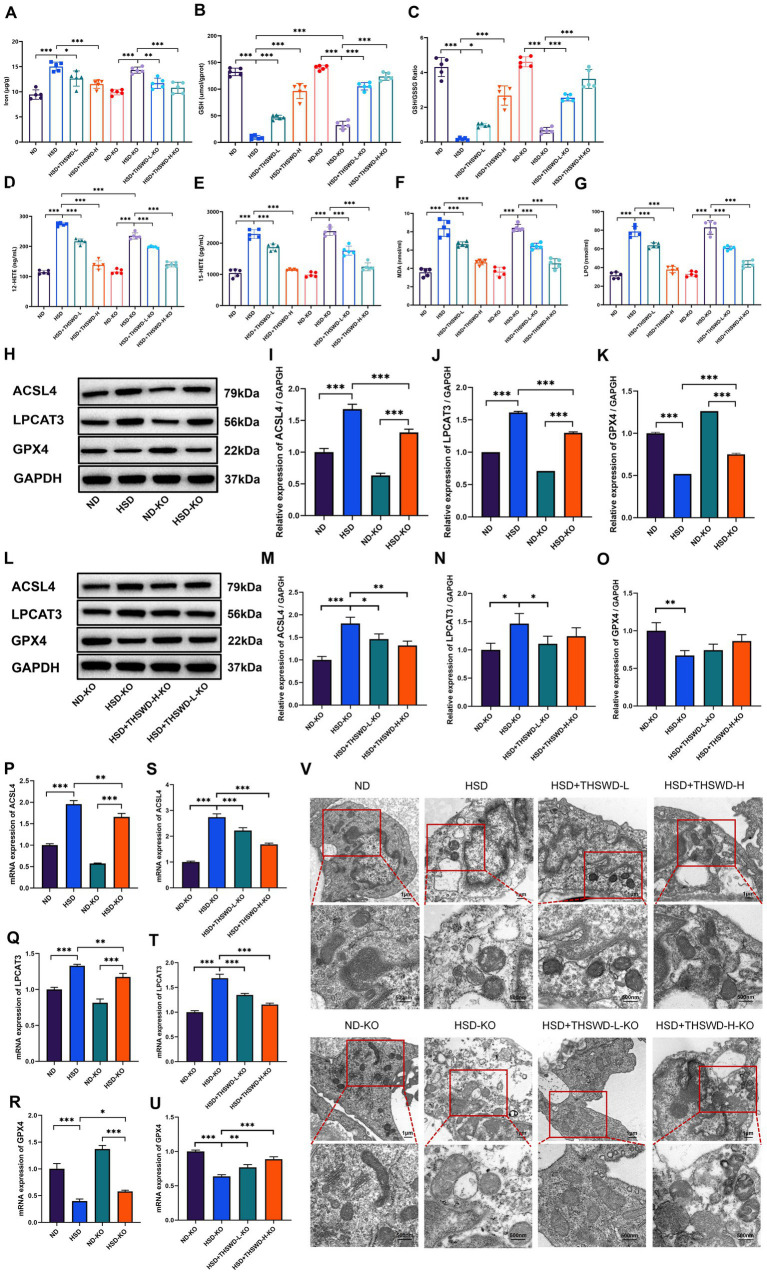
ATF4 knockdown and ATF4 knockdown plus THSWD treatment attenuated ferroptosis in high-salt diet mice. **(A)** Iron levels. **(B)** GSH levels. **(C)** GSH/GSSG levels. **(D)** 12-HETE levels. **(E)** 15-HETE levels. **(F)** MDA levels. **(G)** LPO levels. Data was presented as mean ± SEM (*n* = 5, per group). *p < 0.05, **p < 0.01, ***p < 0.001. **(H–K)** Western blotting results of ACSL4, LPCAT3 and GPX4 in ND, HSD, ND-KO and HSD-KO groups. Data was presented as mean ± SEM (*n* = 3, per group). *p < 0.05, **p < 0.01, ***p < 0.001. **(L–O)** Western blotting results of ACSL4, LPCAT3 and GPX4 in ND-KO, HSD-KO, HSD + THSWD-L-KO and HSD + THSWD-H-KO groups. Data was presented as mean ± SEM (n = 3, per group). *p < 0.05, **p < 0.01, ***p < 0.001. **(P–R)** RT-qPCR results of ACSL4, LPCAT3 and GPX4 in ND, HSD, ND-KO and HSD-KO groups. Data was presented as mean ± SEM (*n* = 3, per group). *p < 0.05, **p < 0.01, ***p < 0.001. **(S–U)** RT-qPCR results of ACSL4, LPCAT3 and GPX4 in ND-KO, HSD-KO, HSD + THSWD-L-KO and HSD + THSWD-H-KO groups. Data was presented as mean ± SEM (*n* = 3, per group). *p < 0.05, **p < 0.01, ***p < 0.001. **(V)** Transmission electron microscopy analysis of aorta tissue. Scale bars, 1 μm and 500 nm. ND, wild-type+normal diet group; HSD, wild-type+high salt diet group; HSD + THSWD-L, wild-type+high salt diet+low-dose Taohong Siwu decoction group; HSD + THSWD-H, wild-type+high salt diet+high-dose Taohong Siwu decoction group; ND-KO, *ATF4^+/−^* + normal diet group; HSD-KO, *ATF4^+/−^* + high salt diet group; HSD + THSWD-L-KO, *ATF4^+/−^* + high salt diet+low-dose Taohong Siwu decoction group; HSD + THSWD-H-KO: ATF*4^+/−^* + high salt diet+high-dose Taohong Siwu decoction group.

ATF4 knockdown significantly reduced aortic levels of 12-HETE and increased levels of GSH (*p* < 0.001). Both high-dose and low-dose THSWD further reversed the abnormal levels of iron, 12-HETE, 15-HETE, MDA, LPO, GSH, and GSH/GSSG (*p* < 0.01; *p* < 0.001) (Figures 8A–G). The knockdown also downregulated the protein and mRNA expression of ACSL4 and LPCAT3 while upregulating GPX4 expression (*p* < 0.05; *p* < 0.01; *p* < 0.001) (Figures 8H–K,P). High-dose and low-dose THSWD additionally downregulated ACSL4 protein and mRNA expression (*p* < 0.05; *p* < 0.01; *p* < 0.001), and low-dose THSWD also reduced LPCAT3 protein and mRNA expression (*p* < 0.05; *p* < 0.001). Although the regulatory effect of THSWD on GPX4 protein expression was not statistically significant, its effect on GPX4 mRNA expression was significant (*p* < 0.01; *p* < 0.001) (Figures 8L–O,S). ATF4 knockdown combined with treatments of both THSWD dosages effectively reversed mitochondrial changes induced by a high-salt diet in mouse aortic endothelial cells (Figure 8V).

### 3.7 Inhibition of ATF4 and THSWD-containing serum inhibit high NaCl-induced calcium overload and ferroptosis in HAECs

**Figure 9:**
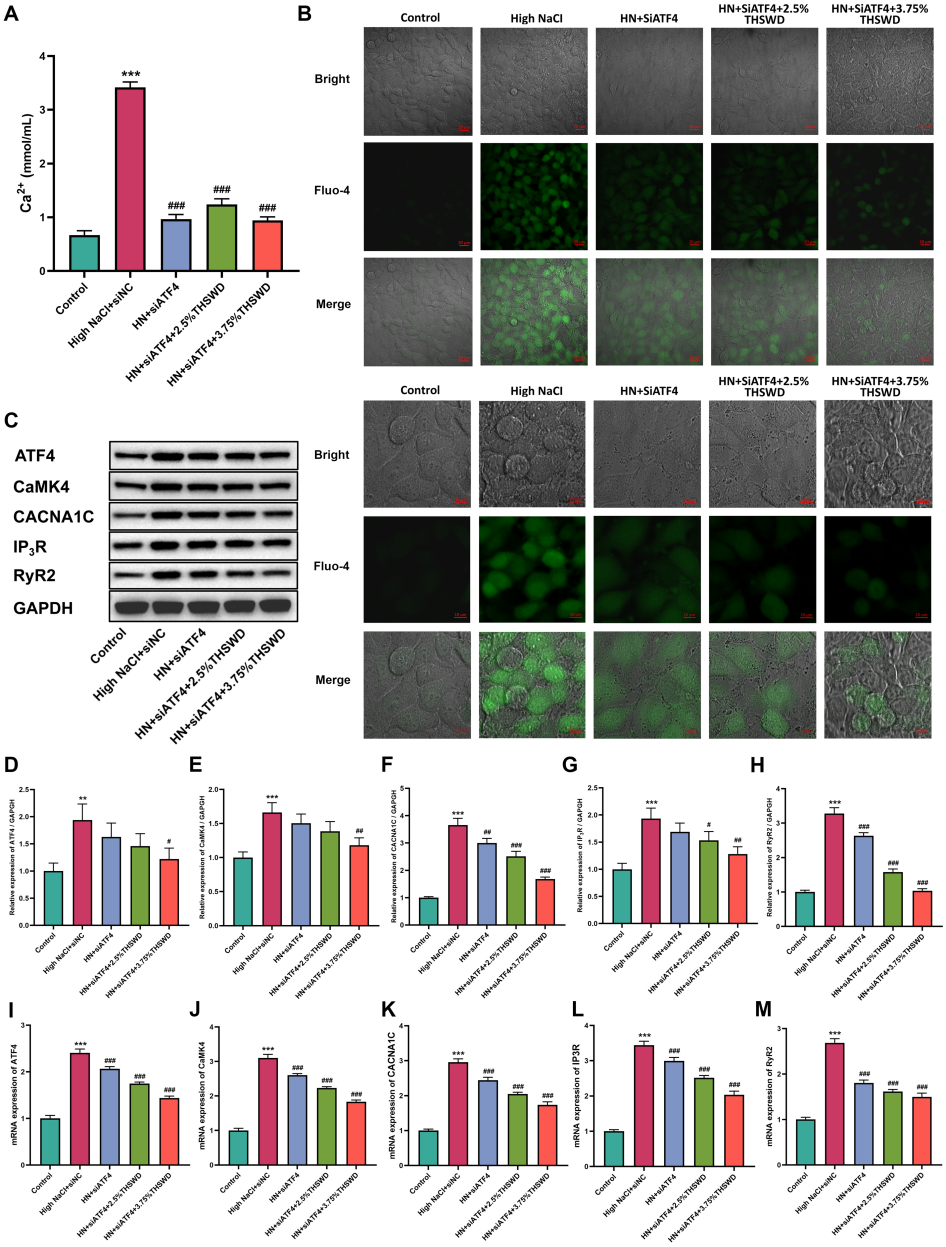
siATF4 and siATF4 plus THSWD-containing serum reduced reduced calcium overload in HAECs. **(A)** Calcium levels. Data was presented as mean ± SEM (*n* = 6, per group). *p < 0.05, **p < 0.01, ***p < 0.001 vs. Control group; #*p* < 0.05, ##*p* < 0.01, ###*p* < 0.001 vs. High NaCl group. **(B)** Confocal fluo-4-Calcium images. Scale bars, 20 μm and 10 μm. **(C–H)** Western blotting results of CaMK4, CACNA1C, IP3R and RyR2. Data was presented as mean ± SEM (*n* = 3, per group). *p < 0.05, **p < 0.01, ***p < 0.001 vs. Control group; #*p* < 0.05, ##*p* < 0.01, ###*p* < 0.001 vs. High NaCl group. **(I–M)** RTqPCR results of CaMK4, CACNA1C, IP3R and RyR2. Data was presented as mean ± SEM (*n* = 3, per group). *p < 0.05, **p < 0.01, ***p < 0.001 vs. Control group; #*p* < 0.05, ##*p* < 0.01, ###*p* < 0.001 vs. High NaCl group. Control, normal group; High NaCl+siNC, High NaCl+siNC negative control group; HN + siATF4, High NaCl+siATF4 group; HN + siATF4 + 2.5% THSWD, High NaCl+siATF4 + low concentration of THSWD-containing serum group; HN + siATF4 + 3.75% THSWD, High NaCl+siATF4 + high concentration of THSWD-containing serum group.

To assess the inhibitory effects of ATF4 and THSWD-containing serum *in vitro*, we transfected HAECs with siATF4 and incubated them with THSWD-containing serum in High NaCI condition. When cells were transfected with siATF4, either alone or combined with incubation with 2.5% or 3.75% THSWD-containing serum, a significant decrease in calcium levels (*p* < 0.001) and reduced green fluorescence intensity were observed (Figures 9A,B). The siATF4 transfection effectively reduced mRNA expression of ATF4 (*p* < 0.001). It also downregulated the protein and mRNA expression of CACNA1C and RyR2 (*p* < 0.01; *p* < 0.001), and significantly decreased the mRNA expression of CaMK4, and IP3R (*p* < 0.001). When cells were incubated with 3.75% THSWD-containing serum, further downregulation of protein and mRNA expression of ATF4, CaMK4, CACNA1C, IP3R, and RyR2 was observed (*p* < 0.05; *p* < 0.01; *p* < 0.001). Incubation with 2.5% THSWD-containing serum downregulated both protein and mRNA expression of CACNA1C, IP3R, and RyR2, while also significantly reducing the mRNA expression of ATF4 and CaMK4 (*p* < 0.05; *p* < 0.01; *p* < 0.001) (Figures 9C–M). The inhibitory effects of 3.75% THSWD-containing serum combined with ATF4 inhibition on these genes and proteins were more pronounced.

**Figure 10:**
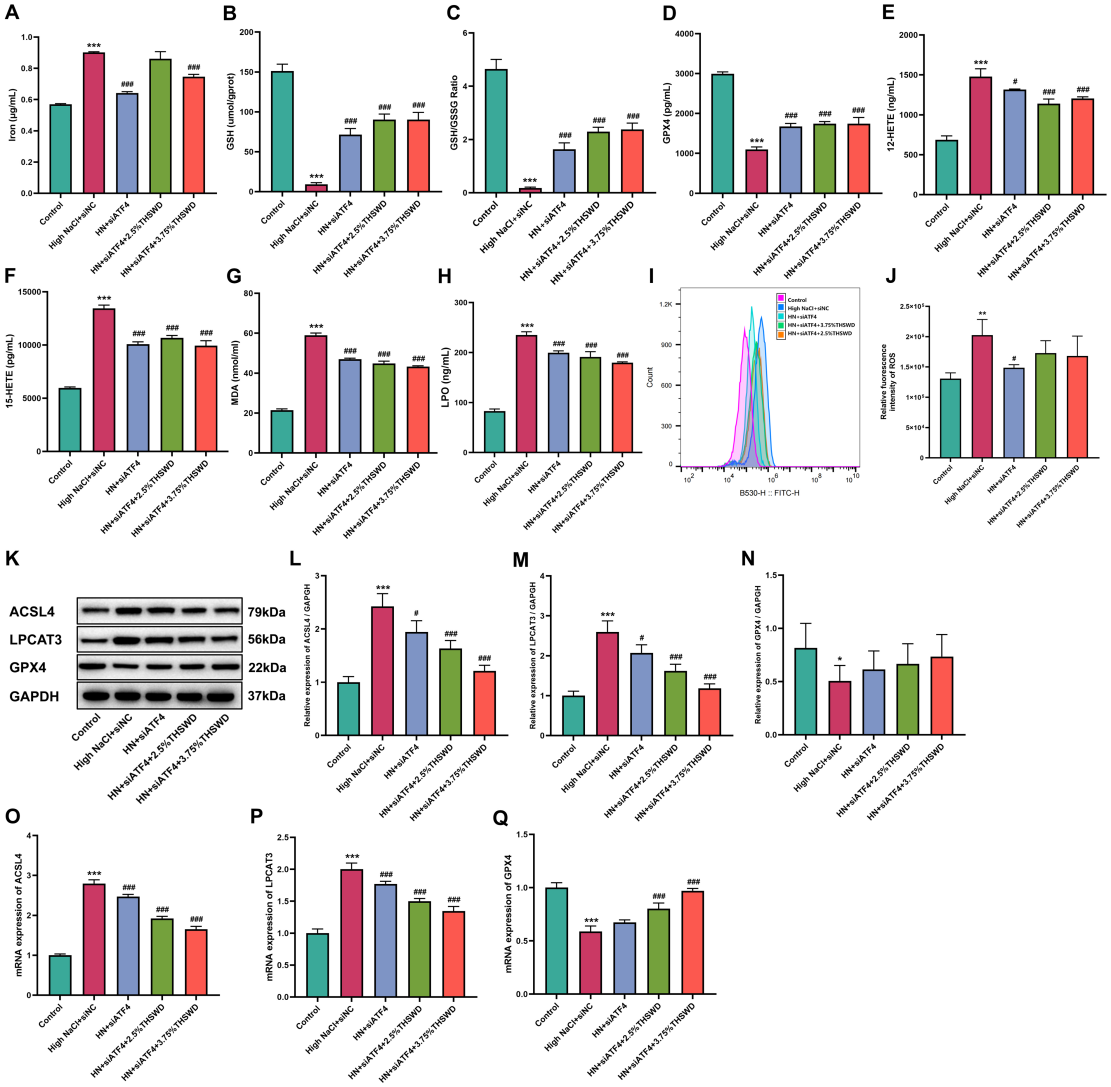
siATF4 and siATF4 plus THSWD-containing serum reduced ferroptosis in HAECs. **(A)** Iron levels. **(B)** GSH levels. **(C)** GSH/GSSG levels. **(D)** GPX4 levels. **(E)** 12-HETE levels. **(F)** 15-HETE levels. **(G)** MDA levels. **(H)** LPO levels. Data was presented as mean ± SEM (*n* = 3, per group). *p < 0.05, **p < 0.01, ***p < 0.001 vs. Control group; #*p* < 0.05, ##*p* < 0.01, ###*p* < 0.001 vs. High NaCl group. **(I,J)** ROS levels. Data was presented as mean ± SEM (n = 3, per group). *p < 0.05, **p < 0.01, ***p < 0.001 vs. Control group; #*p* < 0.05, ##*p* < 0.01, ###*p* < 0.001 vs. High NaCl group. **(K–N)** Western blotting results of ACSL4, LPCAT3 and GPX4. Data was presented as mean ± SEM (*n* = 3, per group). *p < 0.05, **p < 0.01, ***p < 0.001 vs. Control group; #*p* < 0.05, ##*p* < 0.01, ###*p* < 0.001 vs. High NaCl group. **(O–Q)** RTqPCR results of ACSL4, LPCAT3 and GPX4. Data was presented as mean ± SEM (*n* = 3, per group), *p < 0.05 vs. Control group. *p < 0.05, **p < 0.01, ***p < 0.001 vs. Control group; #*p* < 0.05, ##*p* < 0.01, ###*p* < 0.001 vs. High NaCl group. Control, normal group; High NaCl+siNC, High NaCl+siNC negative control group; HN + siATF4, High NaCl+siATF4 group; HN + siATF4 + 2.5% THSWD, High NaCl+siATF4 + low concentration of THSWD-containing serum group; HN + siATF4 + 3.75% THSWD, High NaCl+siATF4 + high concentration of THSWD-containing serum group.

siATF4 led to a significant reduction in levels of iron, 12-HETE, 15-HETE, MDA, and LPO, while increasing GPX4, GSH and GSH/GSSG levels (*p* < 0.05; *p* < 0.001). When cells were incubated with 3.75% THSWD-containing serum, these biomarkers were further decreased (*p* < 0.05; *p* < 0.001), while 2.5% THSWD-containing serum reduced all except iron (*p* < 0.001) (Figures 10A–H). Additionally, siATF4 significantly reduced ROS levels (*p* < 0.05). Although incubation with both 2.5 and 3.75% THSWD-containing serum also lowered ROS levels, these results did not reach statistical significance (Figures 10I,J). siATF4 downregulated the protein and mRNA expression of ACSL4 and LPCAT3 (*p* < 0.05). When cells were incubated with both 2.5 and 3.75% THSWD-containing serum, the protein and mRNA expression of ACSL4 and LPCAT3 were further reduced (*p* < 0.01; *p* < 0.001) and mRNA expression of GPX4 was upregulated (*p* < 0.001) (Figures 10K–Q). The inhibitory effects of 3.75% THSWD-containing serum on these genes and proteins were particularly notable.

## 4 Discussion

This study demonstrated that THSWD significantly ameliorated high salt-induced hypertension and vascular damage by alleviating calcium overload and ferroptosis through ATF4 regulation. *In vivo*, THSWD reversed the adverse effects of a high salt diet, including elevated blood pressure and vascular injury. Similarly, *in vitro* experiments confirmed that THSWD, as well as ferroptosis inhibitors and calcium chelators, improved calcium overload and ferroptosis markers. Notably, *ATF4^+/−^* mice treated with THSWD exhibited superior protective effects on vascular function compared to untreated controls. Collectively, these findings underscore the critical role of ATF4 in modulating the interaction between calcium signaling and ferroptosis, suggesting that targeting this pathway may offer therapeutic benefits in managing salt-sensitive hypertension.

Endothelial dysfunction can lead to increased peripheral resistance through multiple mechanisms, resulting in enhanced arterial constriction and vascular remodeling, ultimately causing hypertension (36). The effectiveness of Chinese herbal medicine for treating hypertension is affirmed (37–39). In this study, we found that THSWD improved calcium overload and ferroptosis in high salt-diet mice, leading to the restoration of blood pressure and endothelial function.

Studies have consistently demonstrated a strong correlation between salt-sensitive hypertension and vascular dysfunction, wherein excessive salt intake weakens vasodilation and disrupts peripheral resistance regulation, ultimately resulting in elevated blood pressure (40, 41). Substantial evidence further indicates that a high-salt diet induces endothelial dysfunction, which plays a pivotal role in the development of hypertension in animal models, including rats and mice (42–45). To assess the impact of a high-salt diet on vascular function and hypertension in mice, we measured serum levels of NO, ET-1, and VEGF as key indicators of endothelial function. Our findings revealed that a high-salt diet significantly decreased NO levels while increasing ET-1 and VEGF levels, accompanied by elevated blood pressure and pathological vascular changes. These results corroborate previous studies, highlighting the detrimental effects of excessive salt intake on vascular health. In addition, elevated cytosolic calcium has been increasingly recognized as a critical hallmark of ferroptosis (46). However, the precise relationship between a high-salt diet, calcium overload, and ferroptosis in the context of hypertension remains poorly understood. In this study, we demonstrated that a high-salt diet induced calcium overload and ferroptosis in hypertensive mice. Importantly, ferroptosis induced by high NaCl in HAECs was effectively inhibited by the calcium chelator BAPTA-AM and the calcium channel blocker NI, showing the central role of calcium in mediating ferroptosis under high-salt conditions.

ATF4 has emerged as a promising therapeutic target for the treatment and prevention of various diseases, with particular relevance in cancer research. While ATF4 expression is generally low in normal cells, it becomes markedly elevated in cancer cells, where it drives pathological processes (47). For example, Yang Wang et al. demonstrated that ATF4 promotes the proliferation, invasion, and migration of gastric cancer cells by transcriptionally activating sonic hedgehog (48). Our previous studies established a hypertension cell model using human umbilical vein endothelial cells (HUVEC). We found that differentially expressed genes were mainly enriched in the ERS signaling pathway, with ATF4 being a key differentially expressed gene. Through miRWalk software prediction, we discovered that miR-1283 might regulate differentially expressed genes related to hypertension, and confirmed that ATF4 is a target gene of miR-1283. These findings have further established a strong association between ATF4 and hypertension (49), showing that ATF4 contributes to the onset of high blood pressure. Specifically, overexpression of ATF4 in mice led to elevated blood pressure and abnormal secretion of vascular factors, whereas ATF4 knockdown in mice fed a high-salt diet prevented blood pressure elevation and improved vascular factor secretion (50). Consistent with these findings, our current study demonstrated that a high-salt diet upregulated ATF4 expression in hypertensive mice, while ATF4 knockdown effectively mitigated salt-induced hypertension, improved the secretion of vascular factors such as NO, ET-1, and VEGF, and reduced vascular damage.

Ferroptosis is a novel form of cell death caused by excessive ROS and imbalanced lipid metabolism, regulated by GPX4. Inhibition of GPX4 activity decreases GSH levels and the GSH/GSSG ratio, leading to ROS accumulation (51, 52). Iron accumulation further accelerates ferroptosis by promoting polyunsaturated fatty acid production in cell membranes, mediated by ferrous iron (Fe^2+^) or lipoxygenase (53, 54). Key enzymes such as ACSL4 and LPCAT3 enhance lipid peroxide accumulation, resulting in excessive ROS production and lipid peroxidation products like LPO and MDA (55–58). Failure of GSH synthesis inactivates GPX4, ultimately causing cell death. ATF4 is involved in oxidative stress, and inhibiting its expression reduces oxidative stress in liver cells (59). Calcium plays a central role in ERS and oxidative stress, with cellular membrane pumps regulating Ca2 + levels (60, 61). The endoplasmic reticulum (ER) releases Ca^2+^ via RyR2 and IP3R, and unfolded proteins in the ER trigger substantial Ca^2+^ release, causing ROS production and oxidative stress (62–65). During ferroptosis, a feedback loop between ROS and Ca^2+^ occurs, where excess ROS damages proteins involved in calcium homeostasis (66). CaMK4 mediates ER-related calcium overload, while CACNA1C is linked to primary hypertension (67, 68).

Despite these findings, the mechanism by which ATF4 regulates Ca^2+^ and ferroptosis in vascular endothelial cells remains unclear. This study explored the relationship between ATF4 and calcium- and ferroptosis-related genes to clarify ATF4’s role in calcium overload and ferroptosis. A high-salt diet induced calcium overload and ferroptosis in hypertensive mice, accompanied by increased ATF4 expression and abnormal calcium and ferroptosis markers, which were rescued by ATF4 knockdown. Similar results were observed *in vitro* using HAECs exposed to high NaCl and siATF4-transfected HAECs. Both *in vivo* and in vitro findings demonstrated that ATF4 promotes calcium overload by regulating CaMK4, CACNA1C, RyR2, and IP3R, and ferroptosis by regulating ACSL4, LPCAT3, and GPX4. Importantly, calcium overload and ferroptosis induced by high NaCl in HAECs were inhibited by ferrostatin-1, BAPTA-AM, NI, and siATF4 transfection. These results highlight ATF4’s pivotal role in calcium overload and ferroptosis in hypertension, suggesting a potential correlation between ATF4, calcium overload, and ferroptosis, consistent with previous studies (69–71).

Traditional Chinese Medicine (TCM) is widely used in China for managing hypertension, often in combination with Western medicine to effectively lower blood pressure in patients with primary hypertension (72). Taohong Siwu Decoction (THSWD), a classic formula known for promoting blood circulation and removing blood stasis, is frequently used in treating hypertension (73). Studies indicate that combining THSWD with antihypertensive drugs is more effective than using a single drug (27). THSWD has been shown to inhibit pro-inflammatory factors and protect endothelial cells (74). In this study, THSWD improved blood pressure and vascular endothelial function while reducing ATF4 and ferroptosis-related protein levels (33). Network pharmacology suggested its mechanism involves calcium signaling pathways (32). We further investigated THSWD’s molecular mechanisms on hypertension, focusing on its impact on ATF4-related calcium overload and ferroptosis. Results demonstrated that THSWD reduced blood pressure in high-salt-induced hypertensive mice and improved vascular dysfunction. It inhibited calcium overload and abnormal ferroptosis-related biomarkers dose-dependently. High-dose THSWD’s effect on calcium overload was comparable to nifedipine, while its impact on ferroptosis-related biomarkers was more potent.

These findings suggest that THSWD may have potential implications for managing salt-sensitive hypertension, a condition that significantly contributes to the global burden of cardiovascular disease (10). Salt-sensitive hypertension is known to involve endothelial dysfunction, inflammation, and calcium signaling abnormalities (75). The ability of THSWD to modulate calcium overload and ferroptosis-related biomarkers observed in this study highlights its potential relevance to these mechanisms. Notably, the effects of high-dose THSWD on calcium overload were comparable to those of nifedipine, while its impact on ferroptosis-related biomarkers appeared more pronounced. The clinical studies also demonstrated that THSWD provides better blood pressure control effects when used alongside existing antihypertensive therapies, with no severe adverse reactions reported (76–78). These findings collectively point to the therapeutic potential of THSWD as a part of the treatment of hypertension.

Given the widespread use of TCM in China and its growing interest globally, THSWD may represent a potential complementary approach worth investigating for salt-sensitive hypertension management, especially in populations with high sodium intake patterns. Salt-sensitive hypertension in Chinese medicine theory is related to blood stasis syndrome, as mentioned in the Miraculous Pivot. According to meta-analyses of hypertension-related TCM constitutions, blood stasis constitution is identified as one of the susceptibility constitutions for hypertension (79, 80). THSWD, known for its effects in activating blood circulation and resolving blood stasis in TCM, plays a role in addressing this susceptibility. Furthermore, its ability to modulate mechanisms associated with high-salt-induced hypertension, such as calcium overload and ferroptosis-related biomarkers. Collectively, these findings suggest that THSWD merits further investigation as a promising therapeutic agent that bridges traditional wisdom with modern pathophysiological understanding in the management of salt-sensitive hypertension.

This study has several limitations that should be addressed in future research. First, while THSWD was shown to regulate ATF4-related calcium overload and ferroptosis, the precise molecular interactions between its active components and ATF4 require experimental verification, additional experimental evidence would enhance our understanding of these interactions. Future molecular studies would help elucidate the specific binding sites and regulatory mechanisms between these compounds and ATF4. Such comprehensive molecular characterization would not only validate our computational predictions but also potentially reveal novel therapeutic targets. Our previous network pharmacology analysis suggested calcium signaling pathways as a key mechanism (32), and experimental approaches such as promoter assays would further characterize these interactions. Second, while the role of ATF4 in hypertension has been demonstrated in previous studies (50), its specific effects on calcium overload and ferroptosis warrant further investigation. While our expression data and computational predictions suggest ATF4’s regulatory role in these processes, future research will establish ATF4 overexpression models in both cell lines and animal studies to definitively evaluate whether THSWD can directly mitigate the negative effects of ATF4 overexpression on calcium overload and ferroptosis. Furthermore, chemical profiling of THSWD using UPLC-Q/TOF-MS has identified several compounds which may contribute to these therapeutic effects in our previous study (33). The multi-component nature of THSWD likely enables synergistic interactions that enhance its overall therapeutic efficacy beyond what might be achieved with single compounds alone. Further research is needed to elucidate the precise mechanisms and clinical applications of this traditional formula. These proposed validation experiments will strengthen our current findings and provide more definitive evidence for the regulatory mechanisms we have identified.

## 5 Conclusion

Taohong Siwu Decoction effectively alleviates high-salt-induced hypertension in mice by improving vascular endothelial function and preserving vascular integrity. Its therapeutic effects are achieved through the regulation of ATF4 in vascular endothelial cells, which inhibits calcium overload and ferroptosis. These findings demonstrate the potential of THSWD as a promising therapeutic strategy for hypertension and its related vascular complications, offering valuable insights into the molecular mechanisms behind its protective effects.

## Data Availability

The raw data supporting the conclusions of this article will be made available by the authors, without undue reservation.

## Supplementary material

SUPPLEMENTARY FIGURE 1 Effect of osmotic control on cell viability in HAECs. Comparison of cell viability between Control, High NaCl (155 mM), and mannitol treatment groups. Data was presented as mean ± SEM (*n*=5, per group). **P*< 0.05, ***P*< 0.01, ****P*< 0.001.

## Glossary

ACSL4: Acyl-CoA synthetase long chain family member 4
ATF4: Activating transcription factor-4
BAPTA-AM: 1, 2-Bis(2-aminophenoxy)ethane-N, N, N′, N′-tetraacetic acid tetrakis(acetoxymethyl ester)
Ca^2+^: Calcium
CaMK4: Calcium/Calmodulin Dependent Protein Kinase IV
CACNA1C: Calcium voltage-gated channel subunit alpha1
CCK-8: Cell Counting Kit-8
CHOP: C/EBP-homologous protein
DR5: Death receptor 5
ELISA: Enzyme-linked immunosorbent assay
eNOS: Enzyme nitric oxide synthase
ER: Endoplasmic reticulum
ERS: Endoplasmic reticulum stress
ET-1: Endothelin 1
Fer-1: Ferrostatin-1
GAPDH: Glyceraldehyde-3-phosphate dehydrogenase
GSH: Glutathione
GSSG: Glutathione disulfide
GPX4: Glutathione peroxidase 4
HE: Hematoxylin–eosin
HUVECs: Human umbilical vein endothelial cells
HAECs: Human aortic endothelial cells
HSD: high salt diet group
IP3R: inositol 1,4,5-trisphosphate receptors
LPCAT3: Lysophosphatidylcholine Acyltransferase 3
LPO: Lipid peroxidation
MDA: Malondialdehyde
ND: Normal diet group
NI: Nifedipine
NO: Nitric oxide
ANOVA: Analysis of variance
RT-qPCR: Real-time quantitative PCR
ROS: Reactive oxygen species
RyR2: Ryanodine receptor 2
SDS-PAGE: SDS polyacrylamide gel electrophoresis
THSWD: Taohong Siwu decoction
THSWD-L: Low dose of Taohong Siwu decoction group
THSWD-H: High dose of Taohong Siwu decoction group
TCM: Traditional chinese medicine
UPR: Unfolded protein response
VEGF: Vascular endothelial growth factor
12-HETE: 12-Hydroxyeicosatetraenoic acid
15-HETE: 15-Hydroxyeicosatetraenoic acid
4-HNE: 4-hydroxynonenal
