# Supplementary figures and images for: Taohong Siwu Decoction alleviates high salt-induced calcium overload and ferroptosis in vascular endothelial cells in hypertension by regulating ATF4
Source: Front Nutr. 2025 Sep 11;12:1647017. doi: 10.3389/fnut.2025.1647017 (PMC12461095; doi:10.3389/fnut.2025.1647017)

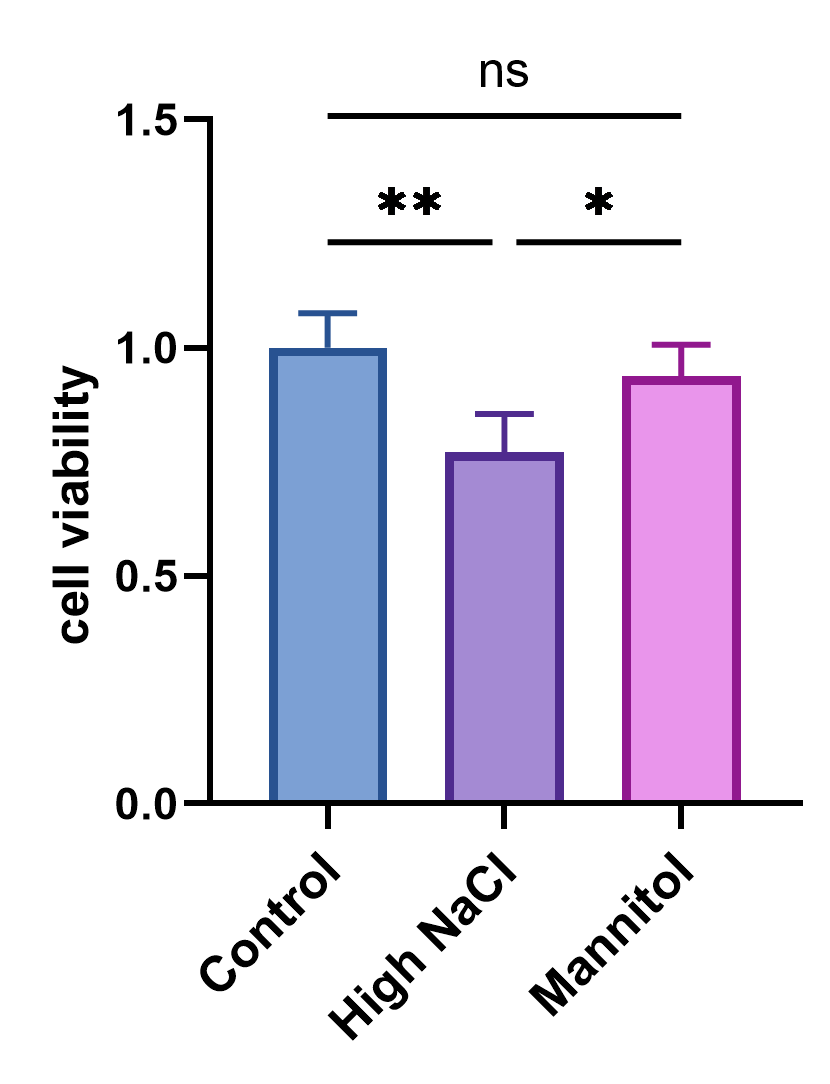

Supplement: SUPPLEMENTARY FIGURE 1 — Effect of osmotic control on cell viability in HAECs. Comparison of cell viability between Control, High NaCl (155 mM), and mannitol treatment groups. Data was presented as mean ± SEM (n=5, per group). *P< 0.05, **P< 0.01, ***P< 0.001. [file Image_1.TIF]
